# Development and Evaluation of a Pseudovirus-Luciferase Assay for Rapid and Quantitative Detection of Neutralizing Antibodies against Enterovirus 71

**DOI:** 10.1371/journal.pone.0064116

**Published:** 2013-06-05

**Authors:** Xing Wu, Qunying Mao, Xin Yao, Pan Chen, Xiangmei Chen, Jie Shao, Fan Gao, Xiang Yu, Fengcai Zhu, Rongcheng Li, Wenhui Li, Zhenglun Liang, Junzhi Wang, Fengmin Lu

**Affiliations:** 1 Department of Microbiology and Infectious Disease Center, Peking University Health Science Center, Beijing, China; 2 Department of Hepatitis Vaccine, National Institutes for Food and Drug Control, Key Laboratory of the Ministry of Health for Research on Quality and Standardization of Biotech Products, Beijing, China; 3 National Institute of Biological Sciences, Beijing, China; 4 Hualan Biological Engineering Inc. , Henan, China; 5 Jiangsu Provincial Center for Disease Prevention and Control, Nanjing, China; 6 The Center for Disease Control and Prevention of the Guangxi Zhuang Automomous Region, Nanning, China; Blood Systems Research Institute, United States of America

## Abstract

The level of neutralizing antibodies (NtAb) induced by vaccine inoculation is an important endpoint to evaluate the efficacy of EV71 vaccine. In order to evaluate the efficacy of EV71 vaccine, here, we reported the development of a novel pseudovirus system expression firefly luciferase (PVLA) for the quantitative measurement of NtAb. We first evaluated and validated the sensitivity and specificity of the PVLA method. A total of 326 serum samples from an epidemiological survey and 144 serum specimens from 3 clinical trials of EV71 vaccines were used, and the level of each specimen's neutralizing antibodies (NtAb) was measured in parallel using both the conventional CPE-based and PVLA-based assay. Against the standard neutralization assay based on the inhibition of the cytopathic effect (CPE), the sensitivity and specificity of the PVLA method are 98% and 96%, respectively. Then, we tested the potential interference of NtAb against hepatitis A virus, Polio-I, Polio-II, and Polio-III standard antisera (WHO) and goat anti-G10/CA16 serum, the PVLA based assay showed no cross-reactivity with NtAb against other specific sera. Importantly, unlike CPE based method, no live replication-competent EV71 is used during the measurement. Taken together, PVLA is a rapid and specific assay with higher sensitivity and accuracy. It could serve as a valuable tool in assessing the efficacy of EV71 vaccines in clinical trials and disease surveillance in epidemiology studies.

## Introduction

Enterovirus 71 (EV71), a single, positive-stranded RNA virus that belongs to the Enterovirus genus of the picornaviridae family, is a highly neurotropic virus; it has been regarded as the most serious neurotropic enterovirus following the eradication of the poliovirus [1], [2]. As one of the major causative agents for hand, foot and mouth disease (HFMD), EV71 is often associated with severe central nervous system diseases, including aseptic meningitis, brainstem and/or cerebellar encephalitis and acute flaccid paralysis [3]. Since first being isolated in California, USA in 1969 [4], as a major pathogen, EV71 has been associated with several high-mortality epidemics: in Bulgaria 1975 (44 deaths), in Hungary 1978 (47 deaths), in Malaysia 1997 (at least 31 deaths), and in Taiwan 1998 (78 deaths) [5]. From 2008 to 2012, there were over seven million cases of HFMD reported in Mainland China, leading to 2,443 deaths [6]. There is no effective antiviral treatment for severe EV71 infections. Therefore, developing vaccines against EV71 is of high priority. Currently, one vaccine candidate entering phase I clinical trials is in Taiwan and Singapore, respectively, while in mainland China, three candidate vaccines currently have been under phase III clinical trials [7].

The presence of neutralizing antibodies against EV71 indicates the protective immunity acquired after EV71 infection [8], [9]. Serum EV71-NtAb can be quantitatively determined by the traditional CPE based neutralization test. However, the CPE method is labor-intensive and time-consuming; it usually takes 5–7 days to obtain a result [10], [11]. A recently reported ELISA (Enzyme-linked Immunosorbent Assay) method for detecting the concentration of EV71 NtAb shortened the experimental procedure to 40 h [12]; however, this method failed to generate accurate quantitative results due to the lack of standard antibody titer. Neutralization assays based on the suppression of reporter gene expression have been widely used for determining the levels of NtAb against enveloped viruses (e.g. HIV and influenza viruses) [13], [14], [15], [16]. In the pseudovirus luciferase assay (PVLA), the inhibition of viral entry into cells by NtAb is correlated to the decreased levels of luciferase signals in the cells. This method is superior to the conventional assay because of its simplicity, higher sensitivity and accuracy, suitability for high-throughput experiments. In addition, no live virus is used during the test. Pseudovirus luciferase assay has not yet been reported for the quantitatively detection of NtAb against EV71. Here, we reported the development of a pseudovirus luciferase assay-based EV71 NtAb measuring system, the primarily use of this system to measure the participants' serum EV71-NtAb levels after receiving candidate EV71 vaccine inoculation, from 3 clinical trials. Our data demonstrated that this system is valuable for the analyses of clinical samples from epidemiology studies and vaccine evaluation.

## Materials and Methods

### 1. Cells and virus

Human rhabdomyosarcoma (RD) cells (ATCC, CCL-136, a gift from the National Vaccine & Serum Institute) were maintained in minimum essential media (MEM, GIBCO BRL) containing 10% fetal bovine serum and 1% HEPES (GIBCO BRL) at 37°C with 5% CO_2_. MEM was used as diluent for the preparation of samples, virus and pseudotypes. EV71 strain 523-07T (Genbank accession no. EU703812) of subtype C4, isolated from a patient in Fuyang city during the HFMD epidemic of 2007 in China, was the viral strain used in our CPE-based neutralization assay [17], [18]. This strain was also used for the construction of pseudotypes and for propagation and titration on RD cells.

### 2. Clinical serum samples

A total of 144 serum samples were collected from 72 individuals who took part in one of the three clinical studies of inactivated EV71 vaccines from different manufacturers (Beijing Vigoo Biological Co., Ltd. Clinicaltrials.gov ID: NCT01313715; Chinese Academy of Medical Sciences, Clinicaltrials.gov ID: NCT01391494; Sinovac Biotech Co., Ltd, Clinicaltrials.gov ID: NCT01273246). Serum specimens of pre-immunization and of 28 days after immunization from each individual were collected. There was roughly a 1∶1 male and female ratio in each group of participants. Written informed consent was obtained from parents or guardians of each subject. Independent Ethics Committee approvals were obtained from the Ethics Committee of the Jiangsu Provincial Center for Disease Prevention and Control as well as the Center for Disease Control and Prevention of the Guangxi Zhuang Automomous Region.

### 3. Construction of the EV71 (FY)-Luc pseudotypes

The construction and quantification of the EV71 (FY)-Luc pseudotypes were published previously [19]. Briefly, EV71 replicon was constructed by replacing capsid coding region of EV71 strain 523-07T with a firefly luciferase reporter gene and a T7 promoter at the 5′-end for transcription in vitro. EV71 capsid expresser was constructed to express the structural capsid genes in trans by using pcDNA6.0 vector. EGFP gene was inserted upstream of the EV71 (FY) capsid gene and was used to determine transfection efficiency of the structural genes. EV71 replicon was linearied and used for RNA transcription with RiboMAX large scale RNA production kit (Promega). Purified RNA can be used for transfection or frozen at −80°C until use. EV71 (FY) pseudotype were produced by sequential transfection of capsid expresser and replicon RNA to HEK293T cells. In brief, HEK293T cells in 10 cm dish were first transfected with 15 µg capsid expression construct and then transfected with 10 µg purified replicon RNA at 24 h post transfection. The supernatant containing pseudotype was harvested 24 h post RNA transfection with 2 rounds of freeze-thaw cycle; clarified supernatant was aliquoted and frozen at −80°C until use. EV71 pseudotype was stable at −80°C for at lease six months without significant infectivity decrease. Viral titer was quantified by measuring the genome copy equivalents with real-time PCR (SYBR Premix Ex Taq II (Perfect Real Time), Takara).

### 4. The procedure of PVLA assay

Complements was heat-inactivated at 56°C for 30 min. Two-fold serial dilutions of sera from 1∶8 to 1∶2048 were made and mixed with an equal volume (50 µl) of pseudovirus at 37°C for 2 h in 96-well microtiter plates. Then, 100 µl of RD cell suspension was also added to wells for a final volume of 200 µl. Each plate of samples also included a six-point standard curve (2-fold serial dilution) and three control wells. The plates were then placed in a CO_2_ incubator at 35°C. After incubation, supernatant was discarded and 50 μl of D-luciferin substrate (BD, Beckton Dickinson) was added into each well and allowed to incubate for 10 min in the dark at room temperature. Luciferase activity, expressed in relative light units (RLUs), was determined according to the Luciferase Assay System user's manual (Instrument:Glomax96, Promega, Madison, WI, USA). Antibody concentration was extrapolated using a four parameter logistic fitting algorithm, the standard curve was plotting according to the relationship between the standard-antibody concentration and the luciferase activity. After correcting for dilution, the concentration of the individual values was determined.

### 5. The optimization of PVLA assay

The PVLA assay were optimized in cell density, incubation time, virus content, and the determination of the linear range and fitting algorithm for the standard curve. Cells densities tested included 2×10^4^, 5×10^4^ and 10×10^4^ cells per well. To determine the optimal incubation time for the PVLA, pseudotype virus were incubated with RD cells for 0.5, 4, 8, 12, 16, 20, and 24 h. To determine the optimal viral inoculum dose for the assay, both the natural infectivity of the virus in RD cells and the variability of inhibition in the presence of EV71-NtAb standards were considered. A serial dilutions by 2-fold with a total of 12 standard concentrations of EV71-NtAb were assayed. A dose response curve expressed in terms of the response (RLUs on a logarithmic scale) versus the dose (U/ml on a liner scale) gives rise to a pronounced sigmoidal shape. The four parameter logistic function fits these data with a high degree of accuracy. In order to estimate the merit of the standard curves, r^2^ and the percentage of the relative error (RE) were determined for each standard point (%RE  =  [(recalculated value – assigned value)/assigned value] ×100%). Existing literature and current guidelines recommend an absolute RE value ≤20% and r^2^ >0.99 [20].

### 6. Reproducibility analysis of PVLA assay

To determine the reproducibility of PVLA assay, two sera, lower (N3) and middle (N12) concentrations of EV71-NtAb, were tested in duplicate on the same plate, on three different days (within a period of two weeks), and by two different certified operators. In total, 24 tests were generated (2 samples ×2 repeats ×3 days ×2 operators  = 24). The reproducibility of PVLA assay was evaluated by calculating the coefficient of variation.

### 7. Cross-reactivity analysis of PVLA assay

To evaluate PVLA cross-reactivity, a variety of control samples were tested: hepatitis A virus antiserum, Polio-I, Polio-II, and Polio-III standard antisera (WHO), goat anti-G10/CA16 serum (NT titer 1280, a gift from the Institute of Medical Biology, Chinese Academy of Medical Science). We selected the CA16 as control for its close gene homology and clinical symptoms to EV71.

### 8. The procedure of CPE assay

The CPE assay was used as “gold standard” to test all samples. In brief, two-fold serial dilutions of inactivated complement sera were mixed with an equal volume (50 µl) of virus working solution containing 100 TCID_50_/well (50% of tissue culture infective dose) of EV71 at 37°C for 2 h in 96-well microtiter plates. 1×10^4^ per well RD cells were added and incubated at 35°C for 7 days. Serum samples were tested in duplicate. The neutralization titer is expressed as the reciprocal of the highest dilution at which over 50% of wells showed complete inhibition of CPE. National reference standard for EV71-NtAb (1000 U/ml) was used as positive control and included in each run. EV71 NtAb was defined as positive if NtAb titers were equal to or greater than 1∶8 dilutions. By using the EV71-NtAb national standard (National Reference Standards No.0024, 1000 U/ml), CPE titers of samples were converted into concentrations with units of U/ml.

### 9. The clinical cutoff of PVLA according to CPE assay

To determine cutoff values for PVLA tests, different cutoffs between 13–17 U/ml were compared with CPE observations. The PVLA method was used to test 326 serum samples selected from a retrospective epidemiological survey (ClinicalTrials.gov ID: NCT01255124) on the occurrence of HFMD in infants with median age of 20 months (ages of 6–35 months), who had participated in the clinical trial titled “The Safety and Immunogenicity of Recombinant Hepatitis B Vaccines in the Healthy Neonates” (ClinicalTrials.gov ID: NCT01183611). The NtAb concentrations measured by the PVLA method were compared against the recorded results of the clinical trial which were determined using the CPE method. A cutoff was chosen when PVLA produces the best overall balance for agreement, sensitivity, specificity with CPE and the highest Youden's index (Y =  Sensitivity + Specificity-1) was obtained. Youden's index has minimum and maximum values of −1 and +1, respectively, with a value of +1 representing the optimal value for an algorithm and specificity with CPE.

### 10. Correlation analysis between PVLA and CPE assay

In addition, regression and Bland-Altman analyses using double-positive samples were performed to evaluate the correlation between both assays. Bland-Altman analysis was typically used to compare measurement techniques against a reference value. The Bland-Altman graph plotted the difference between two techniques against their averages. Statistical evaluation was conducted using the Graph Pad Prism 5.0.

## Results

### 1. Optimization of pseudovirus luciferase assay

**Figure 1:**
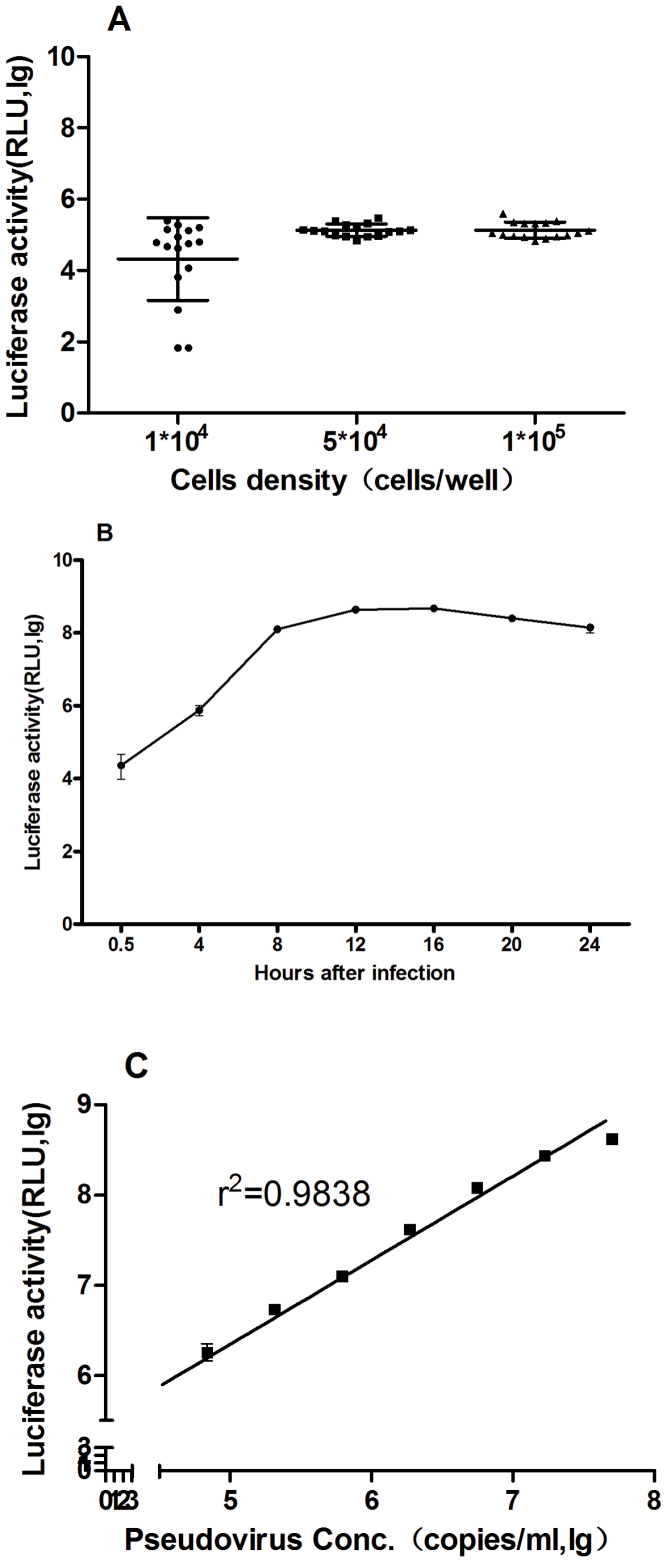
Optimization of assay parameters. (**A**) **Determination of RD cell density.** RD cells (5×10^4^ cells per well) were incubated in a 96-well plate for 12 h with various dilutions of pseudovirus (from 2×10^3^ to 2×10^7^ copies per well) in a final volume of 200 μl per well. The data showed that the RLUs decreased with pseudotype in a dose dependent manner. The variation coefficient was lower than 5% for 5×10^4^ and 1×10^5^ cells per well at all the doses of pseudovirus. However, when the RD cell density was 2×10^4^ cells per well, the variation coefficient of RLU was higher than 15% with lower pseudovirus dosage. So the 5×10^4^ cells per well was chosen as the optimized RD cell density. (**B**) **Evaluation of luciferase activity at various incubation times.** RD cells (5×10^4^ cells per well) were incubated with 10^6^ copies pseudovirus per well in a 96-well plate for various time points, from 0.5 to 24 h, and each luciferase reading shown is the average of eight replicates. The results indicated that luciferase activity increased gradually with the extension of the incubation over time and reached a plateau at 12 h after incubation. therefore, 12 h was chosen as the optimized incubation time. (**C**) **Infection curve of EV71(FY)-Luc.** RD cells were incubated at 35°C together with a range of EV71 pseudovirus concentrations in a final volume of 200 μl per well in 96-well plates. Luciferase activity was measured 12 h post-infection. Each data point represents the average of eight replicates. Linear regression analysis was conducted using Graph Pad Prism. The results showed that when the dose of pseudovirus were within the range of 1×10^4^–10^7^ copies per well, there was a strong correlation between the viral dose and luciferase activity.

For optimization of RD cell density, cells with various densities (including 2×10^4^, 5×10^4^ and 1×10^5^ cells per well) were cultured with serial diluted pseudovirus for 12 h at 37°C. The cell-pseudovirus mixture was present in 16 replicate wells. The data showed that the RLUs decreased with pseudotype in a dose dependent manner. The variation coefficient was lower than 5% for 5×10^4^ and 1×10^5^ cells per well at all the doses of pseudovirus. However, when the RD cell density was 2×10^4^ cells per well, the variation coefficient of RLU was higher than 15% with lower pseudovirus dosage (Figure 1.A). So the 5×10^4^ cells per well was chosen as the optimized RD cell density.

Using the three parameter equation obtained above, we next established the response curve between antibody concentration and luciferase activity. The linear range of neutralization antibody concentration was determined from 0–12.5 U/ml (r^2^ >0.99). Based on this observation, six antibody concentrations (0, 0.62, 1.2, 2.5, 5.0, and 10.0 U/ml) were selected for construction of the standard curve. The concentration of antibody in samples was estimated by fitting the measured luciferase activities to the standard curve.

### 2. The reproducibility of PVLA assay

The two quality control sera containing a low and a middle concentration of EV71-NtAb were measured in duplicate by two operators at three different times. Results showed that the CV values measured by the PVLA method were 9.4% and 11.1%, respectively (Table S1), which demonstrated that the reproducibility of PVLA met the requirement of EV71-NtAb assays.

### 3. The Cross-reactivity of PVLA assay

A variety of samples including hepatitis A virus antiserum, Polio-I, Polio-II, Polio-III standard antisera, and goat anti-G10/CA16 serum were used to evaluate the cross-reactivity of PVLA. As shown in Table S2, the neutralizing antibody titers measured by CPE were lower than 1∶8 and lower than 15 U/ml by PVLA, implied no cross-reactivity of this assay.

### 4. The cutoff value and the agreement of PVLA assay with CPE

**Figure 2:**
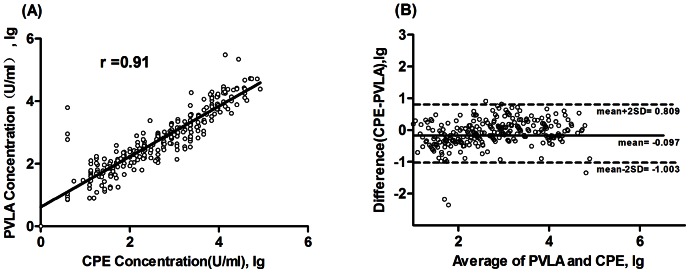
Comparison between CPE and PVLA measurements. (A) Correlation analysis was performed for 180 double-positive EV71-NtAb samples tested by both the CPE and PVLA methods. The solid line represents the linear regression fitting of the data. The result demonstrated that there was a good relativity between the two assays (r = 0.91, p<0.0001). (B) A Bland-Altman difference plot of EV71-NtAb measurements was constructed using the results of the CPE and PVLA methods. The x-axis corresponds to the average (log_10_) concentration of EV71-NTAb determined by CPE or PVLA, and the y-axis is a measure of the difference between the concentrations as determined by CPE and PVLA. The solid line represents the mean value, while dashed lines represent the 95% confidence limits. The results indicated that the new method has a high-degree of agreement with the “gold standard” assay. Only four values fell outside ±2 standard deviations (−1.003 to 0.809log_10_).

Youden's index was used to estimate the agreement of the two methods. As shown in Table 1, the highest Youden`s index was obtained when PVLA NtAb cutoff was fixed at 15 U/ml, a level which produced the best balance of overall agreement, sensitivity, and specificity with respect to the reference CPE assay. At this cutoff value, the overall agreement was 97.2% (95% CI between 94.8–98.7%), with a sensitivity and a specificity calculated at 98.4% (95% CI between 95.3–99.7%) and 95.8% (95% CI between 91.1–98.4%). The results of positive predictive value (PPV) and negative predictive value (NPV) were 97% and 98% respectively. (Tables 1 and 2). Next, the quantitative results of 180 double-positive samples measure by CPE and PVLA methods were compared (Figure 2). The result demonstrated that there was a good correlation between the two assays (r = 0.91, p<0.0001) (Figure 2A). Further Bland-Altman difference analysis also showed that the results from the two methods are highly consistent (97.8%, 176/180). The average of logarithmic difference for quantitative results of the two methods was −0.097, and the standard deviation was 0.093. Only four values fell outside ±2 standard deviations (−1.003 to 0.809log_10_) (Figure 2B). These results indicate that the new method has a high-degree of agreement with the “gold standard” assay.

**Table 1:** Contingency table between PVLA and the CPE assay using different cutoffs^a^.

Antibody concentration cutoff(U/ml)	Agreement(95%CI)	Sensitivity(95%CI)	Specificity(95%CI)	Youden's index
13	0.966(0.942–0.982)	0.989(0.961–0.999)	0.937(0.884–0.971)	0.926
14	0.969(0.944–0.985)	0.984(0.953–0.997)	0.951(0.902–0.980)	0.935
15	0.972(0.948–0.987)	0.984(0.953–0.997)	0.958(0.911–0.984)	0.942
16	0.969(0.944–0.985)	0.978(0.945–0.994)	0.958(0.911–0.984)	0.936
17	0.963(0.937–0.981)	0.967(0.930–0.988)	0.958(0.911–0.984)	0.925

a n = 326.

**Table 2:** Calculated specificity, sensitivity, and positive and negative predictive values for the PVLA in comparison to the CPE.

		CPE	
	n = 326	+	−
Pseudo	+	180	6
	−	3	137
Sensitivity		98.4%(95%CI:95.6–99.6%)	
Specificity		95.8%(95%CI:91.5–98.3%)	
PPV		97%(95%CI:93.4–98.7%)	
NPV		98%(95%CI:94.3–99.4%)	

PPV: Positive predictive value; NPV: Negative predictive value.

### 5. Clinical application of PVLA assay

**Figure 3:**
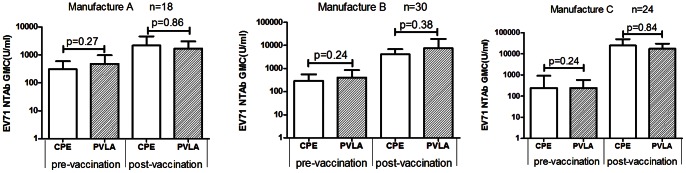
Comparison of EV71-NTAb concentration between CPE and PVLA measurements. Sera were obtained from phase I clinical trials of inactivated EV71 vaccine which were provided by three vaccine manufacturers. NtAb concentration(U/ml) on day 0(pre-vaccination) and day 28(after the first dose, post-vaccination) were tested by the CPE and PVLA methods. The paired t-test showed that the NtAb concentrations determined by the two detection systems were not significantly different (P>0.05).

A total of 144 samples from EV71 vaccine clinical trials were used to validate the clinical applicability of PVLA assay. These samples were divided into three groups according to the different type of inactivated EV71 vaccines. All samples were detected by both the PVLA assay and CPE assay, and the anti-EV71 geometric mean antibody concentrations (GMCs) measured by these two methods were compared. As shown in Figure 3, the EV71 NtAb concentration of clinical sera could be detected by either PVLA or CPE method in all three sample groups. For 18 paired clinical samples from individuals immunized with vaccine A, the GMCs before immunization were 485 U/ml and 306 U/ml and increased to 1687 and 2176 U/ml after immunization detected by the PVLA assay and CPE assay, respectively. There is no statistically significant difference of GMCs between the two methods in both pre-vaccination and post-vaccination subgroups (P = 0.27 for pre-vaccination and P = 0.86 for post-vaccination) (Figure 3A). Similarly, the GMCs of 30 paired samples from individuals immunized with vaccine B and 24 paired samples from vaccine C also showed no significant differences between PVLA and CPE methods (B vaccine: P = 0.24 for pre-vaccination and P = 0.38 for post-vaccination; vaccine C: P = 0.24 for pre-vaccination and P = 0.84 for post-vaccination) (Figure 3B and Figure 3C). In conclusion, for serum, pre- and post-vaccination, the GMCs measured by PVLA were similar to those obtained by the CPE “gold standard” method.

## Discussion

EV71-NtAb is an important marker for monitoring an EV71 epidemic and assessing vaccine immunogenicity [21], [22], [23], [24]. The evaluation of EV71-induced immunogenicity and prevalence of EV71 infection in epidemiological studies has been largely conducted by using the traditional CPE-based method. Some investigators have been trying to establish new NtAb detection methods [12]; however, an easy-performing method that can be used for the accurate quantificational determination of NtAb titer is still not available.

The establishment of China's national quantitative standards for EV71-NtAb made it possible to quantify the neutralization antibodies titer [25]. Here we showed that using this new PVLA method, the NtAb concentration and luciferase activity give rise to a distinctive s-curve. Also, when the NtAb four-parameter model was fit to our NtAb curve [26], the r^2^ value was >0.99, demonstrating an excellent curve fitting. We found that when the cutoff was set to 15 U/ml for the analysis of the epidemiological survey, the detection sensitivity and specificity was 0.984 and 0.958, respectively. By using the PVLA method we developed, the titer of NtAb could be determined within 12 hours. So we believe this assay is useful for large-scale epidemiological screening because of its high throughput capability [13].

The ideal assay for EV71-NTAb measurement should being able to detect antibodies against different genotypes of EV71, but exhibits no cross-reaction with antibodies against other enteroviruses which have close genetic relationship with EV71, such as Polio, CA16. EV71 can be classified into genotypes A, B, and C [27], [28]. Since 1998, sub-genotype C4 has been confirmed the predominant subtype in mainland China [9], [29], [30], therefore the candidate vaccines and the EV71 strains are all belong to C4 genotype. The PVLA method we developed has no positive reaction with antisera of Polio, CA16, and HAV, but can cross-react with antisera from mice immunized with EV71 genotype A and the genotype C4 virus. Then clinical serum samples were measured in parallel by both PVLA and CPE based methods, results obtained by PVLA method have a good agreement with those of traditional CPE method, this result further confirmed the reliability of the PVLA-based assay for quantitative serum EV71-NTAb measurement. Some reports suggested that there were good cross-reactions between different EV71 genotypes [31], [32]. So, we speculated that this PVLA-based method we developed is valuable in the detection of EV71-NTAb caused by different genotypes, although further evaluation is necessary to determine the potential cross-reactivity for antisera generated by EV71 genotype B.

In summary, here we reported the successful development of a simple, precise, reproducible, time- and labor-saving chemiluminescence-based neutralization assay (PVLA) for the determination of EV71-NtAb titers for human and animal sera. Our preliminary use of this new method was shown to have a good agreement and correlation with the “gold standard” CPE-based method. We expect this PVLA based assay for quantitative EV71-NtAb measurement would be widely used in the clinical evaluation of different type of EV71 vaccines and also in seroepidemiology study.

## Supporting Information

Table S1 **Reproducibility of the PVLA.**
(DOC)

Table S2 **The specific sera against different viruses tested by PVLA.**
(DOC)
