# Supplementary material for: Development and Evaluation of a Pseudovirus-Luciferase Assay for Rapid and Quantitative Detection of Neutralizing Antibodies against Enterovirus 71
Source: PLoS One. 2013 Jun 5;8(6):e64116. doi: 10.1371/journal.pone.0064116 (PMC3673970; doi:10.1371/journal.pone.0064116)
Supplement: Table S1 — Reproducibility of the PVLA. (DOC) [file pone.0064116.s001.doc]

**Table S1. Reproducibility of the PVLA**

| Sample | Mean(U/ml) | SD | *C.V%* |
| --- | --- | --- | --- |
| N3 (positive) | 187 | 18 | 9.4 |
| N12 (positive) | 887 | 98 | 11.1 |
