# Supplementary material for: Development and Evaluation of a Pseudovirus-Luciferase Assay for Rapid and Quantitative Detection of Neutralizing Antibodies against Enterovirus 71
Source: PLoS One. 2013 Jun 5;8(6):e64116. doi: 10.1371/journal.pone.0064116 (PMC3673970; doi:10.1371/journal.pone.0064116)
Supplement: Table S2 — The specific sera against different viruses tested by PVLA. (DOC) [file pone.0064116.s002.doc]

**Table S2.** **The specific sera against different viruses tested by PVLA**

| Sample | Anti-  CA16-1  (1:1280) | Anti-  Polio I  (60U/ml) | Anti-  Polio II  (60U/ml) | Anti-  Polio III  (60U/ml) | Anti-  HAV  (100mIU/ml) |
| --- | --- | --- | --- | --- | --- |
| CPE（titer） | ＜1:8 | ＜1:8 | ＜1:8 | ＜1:8 | ＜1:8 |
| Pseudotype（U/ml） | 6 | 5 | 7 | 3 | 4 |
